# Design, Methods, and Population for a Study of PFOA Health Effects among Highly Exposed Mid-Ohio Valley Community Residents and Workers

**DOI:** 10.1289/ehp.1206450

**Published:** 2013-06-04

**Authors:** Andrea Winquist, Cathy Lally, Hyeong-Moo Shin, Kyle Steenland

**Affiliations:** 1 Rollins School of Public Health, Emory University, Atlanta, Georgia, USA; 2 School of Social Ecology, University of California, Irvine, Irvine, California, USA

**Keywords:** C8 Science Panel, cohort study, perfluorooctanoic acid, PFOA, study design

## Abstract

Background: A cohort of community residents and workers is the basis for a series of epidemiologic studies of a Mid-Ohio Valley population with substantial perfluorooctanoic acid (PFOA) exposure due to releases from a chemical plant.

Objectives: We describe study design, methods, and study participants for a longitudinal cohort study of associations between PFOA exposure and adult chronic diseases.

Methods: Two cohorts were formed, one recruited from community residents who participated in a previous community-wide survey, and one from plant workers. Study participants were interviewed during 2008–2011 regarding demographics, health-related behaviors, and personal history of chronic diseases. Reported diseases were validated through medical records review and registry matching. Here we describe cohort characteristics, compare survey respondents and nonrespondents, provide data on the number of diseases reported and validated, and describe historical estimates of serum PFOA concentrations over time.

Results: The final combined cohort included 32,254 participants (28,541 community; 3,713 worker). Participation rates were high (community, 81.5%; worker, 72.9% of target population). The final population from each cohort was representative of the target population in terms of demographic characteristics and measured serum PFOA concentrations in 2005–2006. The study had a wide exposure range and the number of reported cases of chronic diseases was high, resulting in greater power to detect associations than has been the case for many previous studies.

Conclusions: This is the largest study to date of the health effects of PFOA. The information from this cohort is being used to examine associations between PFOA exposure and multiple adult chronic diseases.

## Introduction

Perfluorooctanoic acid (PFOA, or C8) is a synthetic 8-carbon perfluorinated compound used in manufacturing fluoropolymers such as polytetrafluoroethylene (used as Teflon® nonstick cookware coating) and in products that confer soil, water, stain, and grease resistance (Lau et al. 2007; Prevedouros et al. 2006). PFOA is persistent in the environment, is not metabolized in the body, and has a half-life in humans of approximately 2.3–3.4 years (Bartell et al. 2010; Brede et al. 2010; Olsen et al. 2007). Human exposure to PFOA occurs through food (contamination during preparation and from packaging), drinking water (Shin et al. 2011a, 2011b), house dust (Strynar and Lindstrom 2008), and air (Fromme et al. 2009). PFOA was found in the serum of > 99% of the general U.S. population in the 2003–2004 National Health and Nutrition Examination Survey (NHANES), with a median concentration of 4.0 μg/L (Calafat et al. 2007). Populations living near manufacturing facilities using PFOA have higher serum PFOA concentrations than the general U.S. population [e.g., median, 354 μg/L among 371 residents of Little Hocking, OH (Emmett et al. 2006); geometric mean, 36.9 μg/L among 75 adults in Oakdale, MN (Agency for Toxic Substances and Disease Registry 2008)]. Residential water is thought to be the major route of exposure in these populations (Steenland et al. 2009).

In animals such as mice, rats, and non-human primates, observed effects of PFOA relevant to chronic diseases have included tumors of the testicles, liver, and pancreas; decreases in some immune responses; atrophy of spleen and thymus; hepatomegaly; decreased serum cholesterol levels (in rodents); impaired thyroid hormone homeostasis; increases in estradiol and decreases in testosterone; and weight loss and decreased food consumption (Kennedy et al. 2004; Lau et al. 2007; Steenland et al. 2010). Earlier studies of human health effects of PFOA in relation to adult chronic diseases have been reviewed previously (C8 Science Panel 2012; Steenland et al. 2010) and include occupational mortality and medical surveillance studies and a few community studies. Several studies found relatively consistent positive associations between human serum PFOA concentrations and serum cholesterol (the opposite effect of that seen in rodents) and serum uric acid levels (Steenland et al. 2010). Associations between PFOA and several other outcomes have been inconsistently observed and include positive associations with liver enzyme levels, hypertension, ischemic heart disease and stroke mortality, diabetes mortality, thyroid hormone levels, thyroid disease, osteoarthritis, chronic kidney disease, and some cancers including cancers of the prostate, pancreas, and kidney (C8 Science Panel 2012; Steenland et al. 2010).

Only a few previous epidemiologic studies of PFOA health effects relating to chronic diseases have been longitudinal (Steenland et al. 2010). A few worker medical surveillance studies included multiple time points (Costa et al. 2009; Olsen et al. 2003; Sakr et al. 2007). Two occupational cohort studies examined mortality (Leonard et al. 2008; Lundin et al. 2009), but they had low numbers of deaths and results were inconsistent. One community cohort study examined cancer among participants with overall low exposure levels (Eriksen et al. 2009).

Here we describe a cohort that is the basis for several studies in a series of epidemiologic studies conducted by the C8 Science Panel among a population exposed to PFOA as a result of PFOA releases from a chemical plant in the Mid-Ohio Valley (Frisbee et al. 2009). The C8 Science Panel is a three-person group set up under a 2004 legal settlement to determine whether there is a probable link between PFOA exposure and any human disease.

The chemical plant started using PFOA in manufacturing fluoropolymers in 1951. PFOA was released from the plant in air emissions and as liquid and solid waste released in landfills, on-site digestion ponds, or the Ohio River (Paustenbach et al. 2007). PFOA releases from the plant increased over time, peaked in 1999–2000, and subsequently decreased (Shin et al. 2011a). Details of PFOA releases from the plant and models of the movement of PFOA through the environment have been published (Paustenbach et al. 2007; Shin et al. 2011a). A class action lawsuit filed in 2001 by community members against the chemical company alleged health damage due to PFOA contamination of water supplies. A lawsuit settlement initially funded the C8 Health Project (C8HP), conducted in 2005–2006, which was a survey of people in West Virginia and Ohio whose drinking water had been contaminated with PFOA. The C8HP included a questionnaire (covering demographic characteristics, health-related behaviors, medical history, and residential history), clinical laboratory measurements (e.g., serum levels of cholesterol, liver enzyme levels), and measurement of serum levels of PFOA and nine other perfluorocarbon compounds (Frisbee et al. 2009; Steenland et al. 2009). PFOA was of particular interest because it was known to have contaminated water supplies and was the focus of the lawsuit. To participate in the C8HP, a person had to have been exposed to PFOA-contaminated water from any of six contaminated public water districts or from private water sources in those same geographic areas for ≥ 12 months during 1950–2004 at their residence, workplace, or school. [For the location of the plant and the six contaminated water districts, see Supplemental Material, Figure S1 (http://dx.doi.org/10.1289/ehp.1206450).] C8HP participation rates were high; an estimated 80% of current residents in the six contaminated water districts participated (Frisbee et al. 2009). Approximately 37% of C8HP participants did not live in one of the six water districts at the time of the survey (Steenland et al. 2009). The complete eligible population for the C8HP was not enumerated and no information is available about the overall participation rate or characteristics of nonparticipants. However, estimated participation by water district in the C8HP was not clearly related to PFOA exposure levels (Frisbee et al. 2009).

The legal settlement also created the C8 Science Panel, which built upon the cohort constituted during the C8HP. As part of the C8 Science Panel studies, two cohorts were formed, one recruited from community residents participating in the C8HP and one from plant workers. These cohorts were combined. Here we describe the methods used to form the cohorts, and describe cohort characteristics, including historical estimates of serum PFOA concentrations over time. This is the largest study to date of PFOA health effects, and it is being used to examine the association between PFOA and numerous adult chronic diseases. This study will allow a longitudinal analysis of the association between PFOA exposure and disease, in a population with wide variation in PFOA exposure levels and with a large number of cases of various outcomes.

## Methods

**Figure 1:**
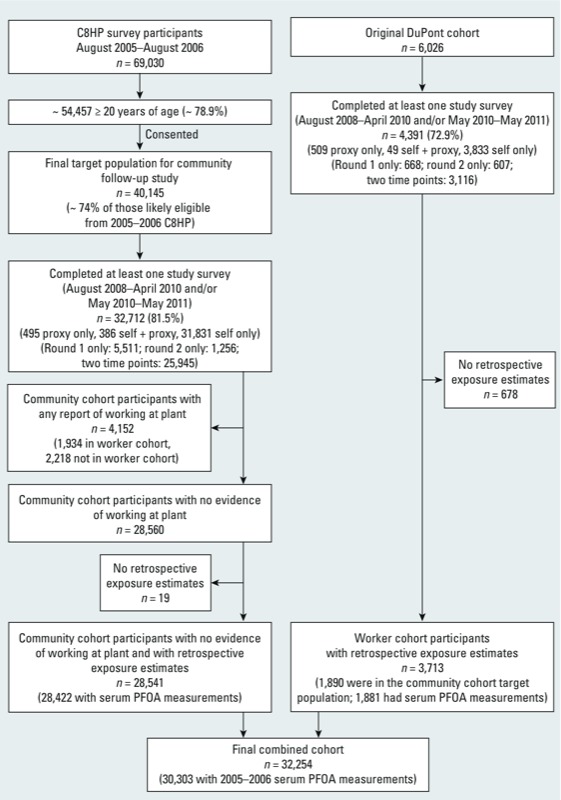
Enrollment of study participants from community and worker cohorts.

*Cohort recruitment*. The overall cohort for this study included a community cohort and a worker cohort, which were combined for analyses. Community cohort participants were recruited among C8HP participants who were ≥ 20 years of age and consented at the time of the C8HP to participate in future C8 Science Panel studies. Only participants ≥ 20 years of age were included because the study focus was on adult chronic diseases. Of the 69,030 C8HP participants, approximately 79% were ≥ 20 years of age and, of these, 40,145 (74%) consented to release of information to the C8 Science Panel, forming the target population for the community cohort recruitment (Figure 1). Worker cohort participants were recruited from a previous occupational cohort consisting of 6,026 persons who worked at the plant between 1948 and 2002 (Leonard et al. 2008), of which 2,090 were also in the community cohort target population.

*Survey administration*. People in the target populations for the community and worker cohorts were asked to complete a survey during August 2008–April 2010 (round 1) that included questions about demographics, health-related behaviors (physical activity, caffeine consumption, smoking, and alcohol consumption), occupational history, residential history, drinking-water consumption, height and weight, and lifetime personal history of various medical diagnoses. Persons who participated in the 2008–2010 survey were asked to complete a follow-up survey during May 2010–May 2011 (round 2) that asked about new medical diagnoses since the time of survey round 1. We also contacted people who had not participated in round 1 and sought to interview them in round 2. Completion of the survey by a proxy was sought for those who were deceased or too ill to complete the survey themselves. Participants or proxies completed the survey either on-line (37% across both survey rounds) or by phone (63% across both survey rounds).

The surveys asked about a person’s history of chronic diseases in several categories (see Table 1 for examples). Participants reporting diseases in each category were asked about the specific type of disease (e.g., type 1 or type 2 diabetes), age at diagnosis and, for some diseases, whether they were currently taking prescription medication or had been hospitalized for the disease. Participants specified the disease type by selecting among specified categories or entering another disease type in a free-text field. Responses to all free-text fields were reviewed and categorized. We cross-checked responses to identify cases of a given disease type that may have been reported in other portions of the survey. If a specific type of disease was reported in any portion of the survey, the respondent was considered to have reported the disease. Women were also asked about reproductive history, including menstrual history, hormone replacement therapy use, and pregnancy history.

**Table 1:** Number of reported cases of adult chronic diseases, final combined cohort (*n* = 32,254).^*a*^

Condition	No. of self-reported cases	No. of self-reported cases for which a medical record was reviewed	No. of validated cases^*b*^	Percent validated among reviewed	No. of validated cases available for prospective analysis
Osteoarthritis	6,641	NA	NA	NA	1,577
Rheumatoid arthritis with medication	1,292	943	317	34	56
Hypertension with medication	12,325	NA	NA	NA	2,319
High cholesterol with medication	9,909	NA	NA	NA	1,898
Coronary artery disease	3,147	2,754	2,502	91	541
Myocardial infarction	1,746	1,479	1,207	82	209
Stroke	1,596	1,245	913	73	269
Diabetes	5,141	4,488	3,651	81	929
Lupus	187	145	72	50	18
Multiple sclerosis	150	102	80	78	14
Thyroid disease with medication	3,027	2,321	2,108	91	467
Chronic kidney disease	725	557	433	78	169
Liver disease	1,896	1,251	715	57	267
Ulcerative colitis	596	419	151	36	29
Crohn’s disease	178	126	95	75	13
Parkinson’s disease	144	115	83	72	36
Chronic obstructive pulmonary disease	3,755	2,732	1,668	61	540
Asthma with medication	2,239	1,657	1,299	78	219
Malignancies (any of 21 sites)	3,292	2,555	2,361	92	^*c*^
NA, not available.^******^^***a***^Analyses of specific diseases reported elsewhere using these data may have applied exclusion criteria or specific case definitions that were different from those used here, leading to differences between numbers listed here and reported elsewhere. ^***b***^Validation was through medical records review or registry matching (for cancer and chronic renal disease). Validation was not sought for osteoarthritis, hypertension, or high cholesterol. Among participants reporting diseases for which validation was sought, ~75% consented to medical record review; among those who consented, a record was obtained for 92%. ^***c***^The number of validated cancers available for prospective analysis varied by cancer type.					

*Supplemental information from the C8HP in 2005–2006*. Most of the data used for this study were new data gathered in 2008–2011 rather than data from the earlier C8HP (2005–2006). However, when information about demographic characteristics (e.g., sex, race, years of education), health-related behaviors (e.g., smoking), health-related characteristics [e.g., body mass index (BMI)], reproductive history, and residential history was missing from the 2008–2010 and 2010–2011 surveys, information from the 2005–2006 C8HP was used when comparable information was available. Information about family history of specific diseases and validation of specific medical conditions (based on C8HP medical record reviews) was also obtained from the C8HP for several outcomes. In addition, PFOA serum concentration measurements in 2005–2006 were obtained from the C8HP.

*Disease confirmation from external sources*. Participants reporting a history of selected medical diagnoses [see Supplemental Material, Table S1 (http://dx.doi.org/10.1289/ehp.1206450)] on the 2008–2010 or 2010–2011 surveys were asked to provide consent for the release of relevant portions of their medical records. We validated reported cases of selected chronic diseases through review of medical records from providers specified by the participant or through matching with state cancer registries (for reports of cancer) or the U.S. Renal Data System (2012) (for cases of chronic renal disease). Medical record reviews were performed by trained medical record–abstractors. Matching with the National Death Index (Centers for Disease Control and Prevention 2012) was also conducted.

*Retrospective serum PFOA concentration estimates*. Retrospective serum estimates for community residents were obtained from a multistage modeling procedure described in detail elsewhere (Shin et al. 2011a, 2011b). Briefly, an environmental fate and transport model was used to generate yearly estimates, starting in 1951, of PFOA concentrations in local air, surface water, and groundwater. These estimates were based on historic emission estimates from the plant, the physicochemical properties of PFOA, and local geologic and meteorological data (Shin et al. 2011a). To estimate each person’s yearly PFOA intake rate in a community exposure model, the estimates of air and water PFOA concentrations from the fate and transport model were used in combination with residential history information from the C8HP and the 2010–2011 survey, information about drinking-water sources and water consumption rates, and public water supply network maps (Shin et al. 2011b). Finally, a pharmacokinetic model was used to generate yearly serum PFOA concentration estimates based on each person’s yearly intake rate estimates, demographic information, self-reported body weight, estimated background exposures, and PFOA half-life estimates (Shin et al. 2011b).

Job and department-specific estimates of yearly PFOA serum concentrations were predicted for the worker cohort using 2,125 historical serum PFOA measurements, knowledge of process changes, and each participant’s work history (Woskie et al. 2012). Yearly serum estimates from the occupational exposure model were used for the years when people worked at the plant if they were higher than residential estimates; if they were lower, the residential (community) estimates were used. For approximately 82% of workers, yearly occupational exposure estimates were always higher than corresponding residential estimates. For the years after working at the plant, serum estimates were decayed at a rate of 18% per year, based on a presumed half-life of 3.5 years (Olsen et al. 2007), until they reached a level predicted by the model for community residential exposure. A half-life of 3.5 years was used for this purpose because this half-life was used in the community pharmacokinetic model, and it yielded estimates closer to the measured values than estimates derived using a shorter half-life (Shin et al. 2011b).

Because plant emissions decreased by 99% between 2000 and 2006 and granular activated carbon filtration of drinking water had been implemented in all municipal water systems by the end of 2008 (Shin et al. 2011a, 2011b, 2012), exposure was assumed to have ceased; therefore, model-based estimates of serum concentrations were generated through 2008 only. Serum concentration estimates for 2009–2011 were generated assuming a constant decay rate of 18% per year, again based on an assumed half-life of 3.5 years (Olsen et al. 2007).

For studies of individual outcomes, primary exposure–response analyses use yearly unadjusted modeled serum estimates or a cumulative exposure metric, calculated as the sum of all unadjusted serum concentration estimates from birth through a given year. For sensitivity analyses, a set of estimates was also generated using a Bayesian calibration procedure that adjusted serum estimates based on the 2005–2006 measurements, weighting measurements more heavily in the years closer to the time of the measurements. An additional set of estimates was generated based on plant emissions only (without adding estimated background exposure).

Persons with insufficient information for generation of retrospective serum PFOA concentration estimates were excluded from the cohorts. Of the 4,391 workers who responded to at least one study survey, 678 were excluded due to lack of occupational exposure estimates or insufficient residential history information (Figure 1). In addition, 2,218 community cohort members who reported working at the plant but were not in the worker cohort, and therefore did not have plant work histories, were excluded because serum PFOA concentrations could not be accurately estimated for these people without occupational exposure estimates. An additional 19 community cohort members were excluded due to insufficient residential history information (Figure 1).

To examine associations between time-varying serum PFOA concentration estimates and personal characteristics, we modeled the natural log of yearly serum PFOA concentration estimates as a linear function of sex, race, 10-year birth year category, time-varying smoking status and alcohol consumption, and non–time-varying household income and years of education at the time of the first survey during 2008–2011 (socioeconomic status markers), BMI, and plant worker status while controlling for time trends and accounting for correlations between repeated measures on the same participant. These analyses were conducted using SAS PROC GENMOD (version 9.2; SAS Institute Inc., Cary, NC).

## Results

*Cohort recruitment*. Study participant enrollment is illustrated in Figure 1. Of the 40,145 people in the community cohort target population, 32,712 (81.5%) completed at least one study survey. Of the 6,026 people in the worker cohort target population, 4,391 (72.9%) completed at least one study survey. Exclusions from both cohorts are listed in Figure 1. A total of 32,254 participants were included in the final combined cohort. Overall, the median length of follow-up after age 20 was 32.9 years, with slightly longer follow-up among workers (median 39.2 years) than among community cohort members who did not work at the plant (median 32.0 years). The median length of follow-up after age 20 that occurred after a person was first known to have lived in one of the six water districts or worked at the plant was 24.0 years. The median length of follow-up after the time of the C8HP (or after 1 August 2006 for workers who did not participate in the C8HP) was 4.4 years.

*Demographic characteristics*. Demographic characteristics of the cohorts at the time of the first survey during 2008–2011 are presented in Table 2. Participants did not differ markedly from the target populations (consenting C8HP participants ≥ 20 years of age and the original worker cohort). The final cohorts included a lower proportion of persons known to have died than the target populations, especially for workers (who may have died before 2005–2006). Compared with the worker cohort target population, worker survey respondents included a lower percentage of persons in the oldest age groups (born before 1930). Participants in the final cohort who were in the worker cohort (median birth year, 1951) were slightly older than participants in the community cohort who did not work at the plant (median birth year, 1958). Plant workers were also more likely to be male, nonwhite, to have a higher education level, to have never smoked or quit smoking, and to report regular alcohol consumption.

**Table 2:** Community and worker cohort demographics in target and final populations [*n* (%)].^*a*^

	Community cohort		Worker cohort		Final combined cohort
	Target population (*n*=40,145)^*b*^	Included in final cohort [*n*=28,541)^*c*^	Target population [*n*=6,026)	Included in final cohort [*n*=3,713)^*d*^	[*n*=32,254)
Year of birth
<1920	171 (0.4)	114 (0.4)	416 (6.9)	59 (1.6)	173 (0.5)
1920–1929	1,363 (3.4)	944 (3.3)	532 (8.8)	175 (4.7)	1,119 (3.5)
1930–1939	3,813 (9.5)	2,798 (9.8)	873 (14.5)	552 (14.9)	3,350 (10.4)
1940–1949	6,819 (17.0)	5,067 (17.8)	1,276 (21.2)	974 (26.2)	6,041 (18.7)
1950–1959	8,671 (21.6)	6,480 (22.7)	1,109 (18.4)	840 (22.6)	7,320 (22.7)
1960–1969	8,202 (20.4)	5,728 (20.1)	1,166 (19.3)	723 (19.5)	6,451 (20.0)
1970–1979	6,952 (17.3)	4,674 (16.4)	626 (10.4)	376 (10.1)	5,050 (15.7)
1980–1982	4,154 (10.3)	2,736 (9.6)	28 (0.5)	14 (0.4)	2,750 (8.5)
Known to be deceased	1,254 (3.1)	726 (2.5)	1,189 (19.7)	204 (5.5)	930 (2.9)
Female sex	21,146 (52.7)	16,602 (58.2)^*e*^	1,156 (19.2)	758 (20.4)	17,360 (53.8)
Race
White, non–Hispanic	39,083 (97.4)	27,901 (97.8)	NA	3,284 (88.5)	31,185 (96.7)
Other	996 (2.5)	640 (2.2)	NA	134 (3.6)	774 (2.4)
Missing	66 (0.2)	0	NA	295 (7.9)	295 (0.9)
Education
<High school	4,500 (11.2)	3,026 (10.6)	NA	37 (1.0)	3,063 (9.5)
High school	16,650 (41.5)	11,706 (41.0)	NA	1,265 (34.1)	12,971 (40.2)
Some college	13,298 (33.1)	9,441 (33.1)	NA	1,081 (29.1)	10,522 (32.6)
≥College diploma	5,636 (14.0)	4,366 (15.3)	NA	1,328 (35.8)	5,694 (17.7)
Missing	61 (0.2)	2 (0.01)	NA	2 (0.1)	4 (0.01)
Smoking
Never smoked	18,186 (45.3)	13,527 (47.4)	NA	1,989 (53.6)	15,516 (48.1)
Smoked and quit	12,276 (30.6)	8,899 (31.2)	NA	1,297 (34.9)	10,196 (31.6)
Smoked, did not quit	9,683 (24.1)	6,115 (21.4)	NA	427 (11.5)	6,542 (20.3)
Regular alcohol consumption
Never	NA	17,011 (59.6)	NA	1,683 (45.3)	18,694 (58.0)
Yes and quit	NA	4,105 (14.4)	NA	535 (14.4)	4,640 (14.4)
Yes, did not quit	NA	7,360 (25.8)	NA	1,486 (40.0)	8,846 (27.4)
Missing	NA	65 (0.2)	NA	9 (0.2)	74 (0.2)
In community cohort	40,145 (100)	28,541 (100)	2,090 (34.7)	1,890 (50.9)	30,431 (94.3)
NA, not available. ^***a***^Demographic characteristics are as reported on the first survey during 2008–2011, except that information from the 2004–2005 C8HP was used when information was missing from the first survey during 2008–2011. ^***b***^Consented among those in C8HP of qualifying age. ^***c***^The final community cohort included those who had no evidence of working at the plant, responded to at least one survey, and had available serum PFOA concentration estimates. ^***d***^The final worker cohort included those who responded to at least one survey and had available serum PFOA concentration estimates. ^***e***^The percentage female sex was slightly different from the target population due to exclusion of those who worked at the plant, who were predominantly male.					

*Community cohort mobility*. Of the 28,541 persons in the community cohort who were not workers, 3,874 were not known to have ever lived in a qualifying water district (they may have qualified for the C8HP because they worked or went to school in the area). Among the remaining 24,667, the median total duration of known residence in the area was 18.7 years. Of those 24,667 persons, based on available residential history information, 2.8% lived in the area during the entire exposure estimation period (starting in 1951 or at birth, whichever was later; and ending in 2008 or at the last survey date, whichever was earlier) with no gaps > 6 months in length; 1.7% started and ended in the area but had periods when they were not known to have lived in the area; 7.0% started but did not end in the area; 38.6% ended but did not start in the area; and 49.9% neither started nor ended in the area. For periods during which a person was not known to have lived in the area, the exposure model assigned background exposure levels (Shin et al. 2011b).

*Serum PFOA concentration measurements*. Table 3 shows the distribution of measured serum PFOA concentrations from the C8HP in 2005–2006. The median measured PFOA serum concentration among those in the overall cohort was 26.1 μg/L, much higher than background levels in the U.S. population [i.e., 4 μg/L (Calafat et al. 2007)]. Community cohort members had a lower median serum concentration (24.2 μg/L) than workers (112.7 μg/L).

**Table 3:** Serum PFOA concentrations in target and final populations, among those who participated in the C8HP.

	Community cohort				Worker cohort		Final combined cohort(*n*=30,431)
	Target population (*n*=40,145)^*a*^	Respondents to ≥1 survey during 2008–2011 (*n*=32,712)	Respondents to ≥1 survey during 2008–2011 with no evidence of working at the plant (*n*=28,560)^*b*^	Included in final cohort (*n*=28,541)^*c*^	Target population (*n*=2,090)	Included in final cohort (*n*=1,890)^*d*^	
Mean	85.7	89.6	70.9	70.9	317.2	324.6	86.6
SD	262.2	277.6	151.2	151.2	889.4	920.6	278.9
25th percentile	12.8	13.1	12.3	12.3	53.4	55.9	12.8
Median	26.1	27.2	24.2	24.2	109.8	112.7	26.1
75th percentile	68.6	72.3	58.9	58.9	254.6	256.2	68.1
No. with measurements	39,954	32,577	28,441	28,422	2,081	1,881	30,303
^***a***^Consented among those in C8HP of qualifying age. ^***b***^Evidence of working at the plant was based on survey responses to questions about occupational history and working at the plant as well as whether the person was also in the worker cohort. ^***c***^The final community cohort included those who did not have evidence of working at the plant, responded to at least one survey, and had available serum PFOA concentration estimates. ^***d***^The final worker cohort included those who responded to at least one survey and had available serum PFOA concentration estimates.							

**Figure 2:**
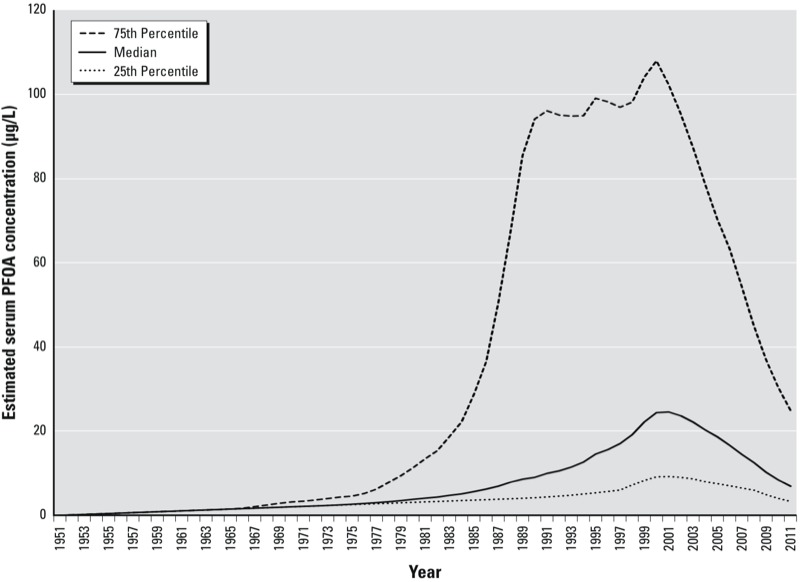
Retrospective serum concentration estimates, by calendar year: final combined cohort.

*PFOA exposure estimates*. Percentiles of the estimated serum PFOA concentrations by calendar year are shown in Figure 2 for the final combined cohort. There was a wide range of estimated serum concentrations. Median estimated serum PFOA concentrations in the overall cohort peaked in 2001. By 2011, median estimated serum concentrations had decreased among both cohorts. Among those in our final cohort who had serum measurements from the C8HP (*n* = 30,303), the Spearman’s rank correlation between measured and estimated PFOA serum concentrations in the year of the C8HP, incorporating both community and occupational exposure estimates, was 0.71. Models of associations between time-varying serum PFOA concentration estimates and personal characteristics [see Supplemental Material, Table S2 (http://dx.doi.org/10.1289/ehp.1206450)] showed associations between higher serum PFOA estimates and earlier birth year, white race, normal or low BMI (vs. higher BMI), working at the plant, former alcohol use (vs. no alcohol use), and female sex (in model controlling for worker status). Compared with those who never smoked, current smokers had lower serum PFOA estimates. There were no clear trends in associations between PFOA exposure estimates and measures of socioeconomic status, including education and household income.

*Reported adult chronic diseases*. The numbers of participants reporting selected adult chronic diseases on the study questionnaire are listed in Table 1. Overall, approximately 60% of participants reported a chronic disease for which medical records were sought. Approximately 75% of these people consented to medical record review; among those who consented, at least one record was obtained for 92%. The percentage confirmed among self-reported cases of disease for which a medical record was obtained varied among conditions (Table 1), being particularly low for rheumatoid arthritis (with apparent poor understanding of arthritis types). Numbers of validated cases available for prospective analyses, considering only new cases after the C8HP (or after 1 August 2006 for workers not in the C8HP), were substantially lower than numbers available for retrospective analyses (Table 1).

## Discussion

This study is the largest longitudinal study to date of a population with high levels of PFOA exposure. The study had high participation rates, and the final population from each cohort was representative of the target population in terms of demographic characteristics and serum PFOA concentrations in 2005–2006. The number of reported cases of chronic diseases was high, resulting in greater power to detect associations than has been the case for many previous studies. A major strength of the study is the availability of information about a wide range of chronic disease outcomes including specific disease types, ages at diagnosis, prescription medication use, and validation of the disease for most outcomes.

The study included people with a wide range of PFOA exposures through inclusion of both community members, including some from less exposed areas, and plant workers. Another major strength of this study is the availability of yearly estimated PFOA serum concentrations for each participant going back to birth or 1951. These estimates correlated well with measured serum PFOA concentrations in 2005–2006.

This study also has several limitations. To be included in the community cohort, a person had to have participated in the C8HP in 2005–2006. Therefore, this is a survivor cohort and has the potential for selection bias for potentially fatal outcomes. The worker cohort target population did not require a person to be alive in a particular year. However, because of difficulties in obtaining information from proxies about persons who had died, the worker cohort also has the potential for survival-related selection bias, as reflected by the lower representation of the oldest groups among survey respondents. The direction and strength of the impact of survivor bias on the analytic results are not clear *a priori*. However, for rare fatal diseases, loss of those with disease would be expected to weaken power. The potential for survivor bias can be partially addressed for some outcomes by comparing the results of analyses including each person’s entire exposure and disease history (starting at 20 years of age or the year 1950) with the results of prospective analyses considering only new disease after 2005–2006 (and excluding those who developed disease before that time), although for some outcomes restriction to these years resulted in small numbers. Furthermore, several outcomes of interest are generally not fatal and would not be expected to be affected by survivor bias.

Case ascertainment based on self-report can lead to outcome misclassification. Our medical records validation allows us to restrict analyses to validated cases of disease, excluding people from the analysis who self-reported disease that was not validated. This restriction can improve the specificity of disease classification. However, because we could not review all medical records for the cohort, we could not detect cases of disease that were not self-reported. Therefore, our analyses could misclassify some diseased people as nondiseased. The percentage misclassified among everyone considered nondiseased is expected to be small and nondifferential with respect to exposure, and this misclassification is expected to have a smaller impact on analytic results than incorrect self-reports of disease.

Another limitation results from the fact that serum PFOA measurements were available only during 2005–2006, but exposure estimates were needed for times in the past. The exposure estimation study used extensive information about plant PFOA releases and residential history to generate the best possible estimates of PFOA exposure over time (Shin et al. 2011a, 2011b). Although correlations between estimated and measured serum concentrations were good in 2005–2006, it is unclear whether correlations would have been different further back in time. Correlations between estimates and actual (but unknown) serum concentrations further back in time could be better due to lower prevalence of unreported bottled water use, but they could be poorer due to factors such as reduced applicability of reported water consumption estimates. The estimation process required some unverifiable assumptions, resulting in uncertainty and variability in retrospective PFOA estimates. Some key assumptions related to water intake. The reliability of information from the surveys regarding tap and bottled water consumption is uncertain, especially for bottled water consumption (Shin et al. 2011b). Other factors that could explain differences between observed and predicted values include locally grown vegetable consumption (which could not be included in the estimation model because data about vegetable sources and consumption rates were unavailable); potential inaccuracies in reported residential histories and in exposure model assumptions (e.g., assumptions about indoor air concentrations, air and water intake rates, and time–activity patterns); and uncertainties in parameters used in models for estimation of water and air PFOA concentrations (e.g., PFOA soil–water partition coefficient) (Shin et al. 2011a). Nevertheless, error in the exposure estimates is likely independent of disease status and would most likely, although not certainly, bias exposure–outcome associations to the null.

## Conclusion

Data from the cohort described here are being used in analyses examining the association between PFOA exposure and a wide variety of adult chronic diseases. This cohort will add to previous information by allowing a longitudinal analysis of the association between PFOA exposure and disease in a population with wide variation in PFOA exposures and with a larger number of cases of the various outcomes than has been possible in many previous cohort studies. The results of analyses from this cohort have been used to support decisions by the C8 Science Panel regarding whether or not there is a probable link between PFOA exposure and human disease (C8 Science Panel 2012).

## Supplemental Material

(332 KB) PDF
