# Supplementary material for: Design, Methods, and Population for a Study of PFOA Health Effects among Highly Exposed Mid-Ohio Valley Community Residents and Workers
Source: Environ Health Perspect. 2013 Jun 4;121(8):893–9. doi: 10.1289/ehp.1206450 (PMC3734501; doi:10.1289/ehp.1206450)
Supplement: (332 KB) PDF [file ehp.1206450.s001.pdf]

Environmental Health Perspectives doi:10.1289/ehp.1206450

Supplemental Material

Design, Methods and Population for a Study of PFOA Health Effects among Highly Exposed Mid-Ohio Valley Community Residents and Workers

Andrea Winquist, Cathy Lally, Hyeong-Moo Shin, and Kyle Steenland

Table of Contents

Supplemental Figure S1: Map of study area 3

Supplemental Table S1: Medical conditions for which medical records review was conducted. . 4

Supplemental Table S2: Parameter values from models of the association between the natural log of yearly modeled serum PFOA concentration estimates and personal characteristics 5

Supplemental Figure S1: Map of study area

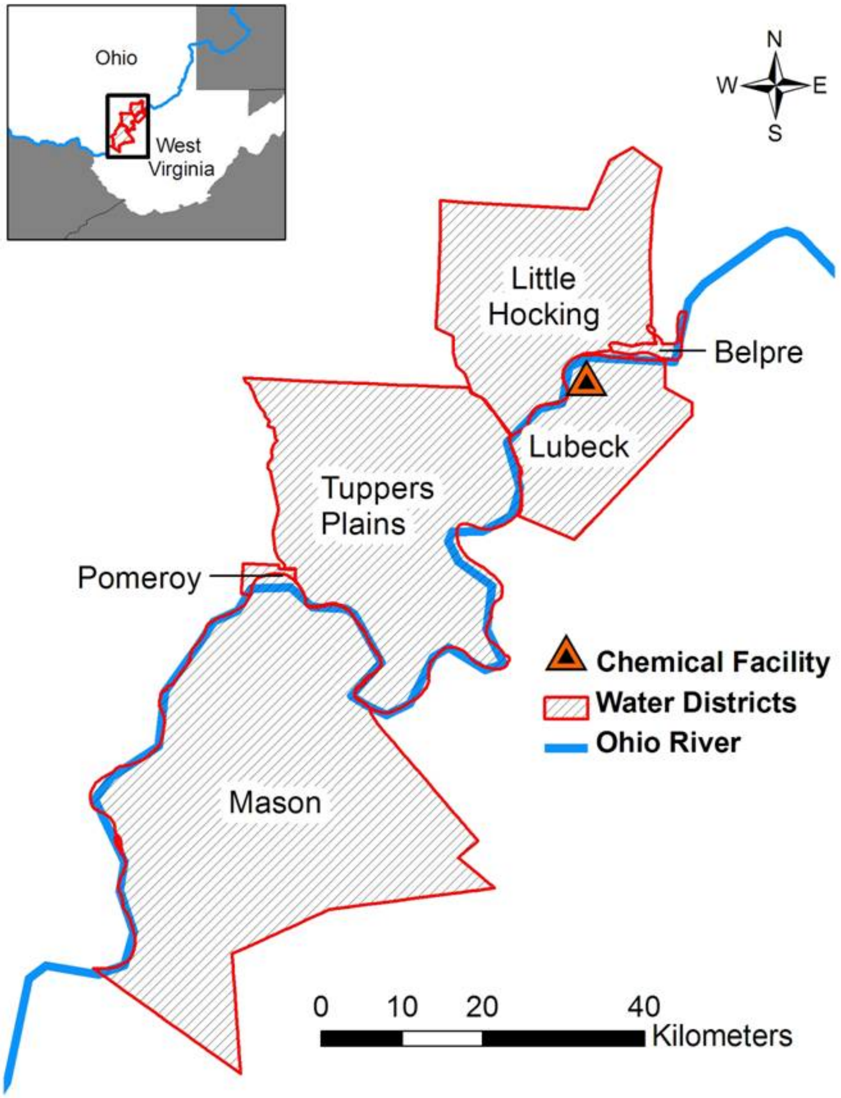

Supplemental Table S1: Medical conditions for which medical records review was conducted.

Condition/Category	Exclusions (medical records not reviewed)
Heart disease	
Cerebrovascular accident	
Diabetes	
Chronic Obstructive Pulmonary Disease (Emphysema or Chronic bronchitis)	
Asthma	No current prescription medication
Thyroid disease	No current prescription medication
Kidney disease	Kidney stones
Liver disease	
Rheumatoid arthritis	No current prescription medication
Autoimmune disease	
Inflammatory bowel disease (Ulcerative colitis or Crohn's)	
Neurological disease	
Cancer	Non-melanoma skin cancer

Supplemental Table S2: Parameter values from models of the association between the natural log of yearly modeled serum PFOA concentration estimates and personal characteristics*

**QIC of full model**	1420627.603
**Parameter estimates:**	
Birth year <1920	**0.5464**
Birth year 1920-1929	**0.3149**
Birth year 1930-1939	**0.2719**
Birth year 1940-1949	**0.1663**
Birth year 1950-1959	ref
Birth year 1960-1969	**-0.1864**
Birth year 1970-1979	**-0.2487**
Birth year 1980+	**-0.2862**
Current smoking	**-0.0185**
Former smoking	-0.0047
Current alcohol use	0.0023
Former alcohol use	**0.0193**
Female gender	**0.0201**
Non-white race	**-0.1322**
BMI underweight	0.0452
BMI overweight	**-0.0754**
BMI obese	**-0.1553**
In worker cohort	**1.0782**
Education: <high school	**-0.0662**
Education: high school	**0.0864**
Education: some college	**0.147**
Education: Bachelor degree +	ref
Income: <$10,000	**-0.1339**
Income: $10,001-$20,000	**-0.0989**
Income: $20,001-$30,000	-0.019
Income: $30,001-$40,000	**0.0552**
Income: $40,001-$50,000	**0.0576**
Income: $50,001-$60,000	-0.0065
Income: $60,001-$70,000	0.0111
Income: $70,000+	0

* Models modeled the natural log of yearly serum PFOA concentrations as a linear function of gender; race; 10-year birth year category; time-varying smoking status (none,current,former) and alcohol consumption (none,current/former); and non-time-varying household income and years of education (socioeconomic status markers), body mass index category, and plant worker status; controlling for time trends using cubic splines with knots at 1961, 1966, 1971, 1976, 1981, 1986, 1991, 1996, 2001, and 2006; and accounting for correlations between repeated measures on the same subject using a 45-dependent correlation structure (mdep(45)). These analyses were conducted using SAS PROC GENMOD. Bold type indicates $p<0.05$.
